# Integrative Transcriptomic Analysis and Co-Expression Network Characterization of Soybean Developmental Tissues

**DOI:** 10.3390/plants15071002

**Published:** 2026-03-25

**Authors:** Dounya Knizia, Khalid Meksem, My Abdelmajid Kassem

**Affiliations:** 1 School of Agricultural Sciences, Southern Illinois University, Carbondale, IL 62901, USA; dounya.knizia@siu.edu (D.K.); meksem@siu.edu (K.M.); 2 Plant Genomics and Bioinformatics Lab, Department of Biological and Forensic Sciences, Fayetteville State University, Fayetteville, NC 28301, USA

**Keywords:** co-expression network, differential gene expression, *Glycine max*, PCA, RNA-seq, seed development, transcriptomics

## Abstract

Soybean (*Glycine max* (L.) Merr.) is a globally important legume crop valued as a major source of plant-based protein and edible oil. Understanding the transcriptional programs underlying tissue-specific development is essential for improving seed quality and agronomic performance. Here, we present an integrative transcriptomic analysis of soybean based on 12 samples representing key seed developmental stages—including globular, heart, cotyledon, embryo, dry seed, mid-mature, and late-mature—and vegetative and reproductive tissues, including leaf, root, stem, flower bud, and seedling at 6 days after imbibition (6 DAI). Following data preprocessing and filtering, 54,880 genes were retained for downstream analysis. Principal component analysis revealed clear separation between seed and non-seed tissues, indicating that tissue identity is the dominant driver of transcriptomic variation. Analysis of the top 100 most variable genes further highlighted distinct expression modules associated with seed maturation and vegetative growth. Differential expression analysis identified 9785 genes exhibiting significant expression differences between seed and non-seed tissues, including 1139 upregulated and 8646 downregulated genes under relaxed statistical thresholds. Functional characterization of seed-upregulated genes revealed enrichment of biological processes related to storage metabolism, embryo development, and stress protection mechanisms associated with desiccation tolerance. In addition, co-expression network and correlation analyses demonstrated strong transcriptional coherence among seed tissues and distinct clustering of vegetative organs. Together, these results provide a comprehensive systems-level overview of transcriptional organization across soybean tissues and identify candidate gene sets relevant to seed biology, functional genomics, and crop improvement.

## 1. Introduction

Soybean (*Glycine max* (L.) Merr.) is one of the most important legume crops worldwide because of its high protein and oil content and its widespread use as a source of animal feed, human food, and industrial raw materials [1,2,3]. Meeting the increasing global demand for plant-based protein and vegetable oil represents a major challenge for modern agriculture and global food security. Transcriptome-wide studies have shown that thousands of genes participate in the temporal and spatial regulation of soybean seed development and maturation [4,5,6]. In parallel, advances in sequencing technologies have enabled the generation of multiple high-quality soybean genome assemblies, greatly expanding the genomic resources available for functional studies [3,7]. Additional genomic tools, including high-density genetic linkage maps and SNP arrays, further facilitate soybean functional genomics and trait dissection [6,8].

Studies of seed development in soybean have revealed that different phases—from globular embryo, heart stage, cotyledon formation, dry seed, to seedling establishment—are characterized by distinct gene expression patterns such as storage protein accumulation, desiccation tolerance, and embryo maturation [1,4,9,10,11]. For instance, early seed developmental stages show high expression of histones and proline-rich proteins, whereas mature dry seeds show elevated levels of late embryogenesis abundant (LEA) proteins and dehydrins [2,10].

These transcriptional programs reflect key physiological transitions underlying embryo development, nutrient accumulation, and maturation. During the globular to cotyledon stage, cellular proliferation dominates and genes related to cell cycle, chromatin remodeling, and transcription factors such as WUSCHEL-related homeobox (WOX) and LEAFY COTYLEDON (LEC) are upregulated [12,13]. As the embryo matures, a developmental shift toward nutrient storage occurs, marked by the activation of genes encoding glycinin, β-conglycinin, and oleosins—essential for protein and lipid deposition in storage vacuoles and oil bodies [14,15]. This storage phase is tightly regulated by hormonal cues such as abscisic acid (ABA), which not only drives seed filling but also induces desiccation tolerance via LEA proteins and antioxidant enzymes [16].

Dry seeds, in contrast, display a highly specialized transcriptome adapted for quiescence and stress protection. Numerous studies report elevated expression of dehydrins, small heat shock proteins, and seed-specific protective proteins like oleosin and caleosin during late maturation [2,17]. These molecules stabilize membranes and proteins during water loss, ensuring viability during desiccation and storage.

Beyond seeds, vegetative tissues (leaf, root, stem, flower bud) also participate in the developmental trajectory of the plant, and comparative transcriptomic atlases across multiple tissues are emerging [18,19]. For example, an integrated transcriptome atlas of soybean organs revealed large-scale gene expression coordination across tissues and developmental stages [18,20]. Vegetative development is characterized by distinct transcriptional signatures aligned with the physiological functions of each organ. For instance, the root transcriptome is enriched in genes involved in water and nutrient uptake, stress response pathways, and hormone biosynthesis such as auxin and cytokinin, which play crucial roles in root growth and lateral branching [1,21]. In contrast, the shoot apex and stem tissues activate meristem maintenance genes and regulators of vascular tissue differentiation, such as HD-ZIP III and NAC-domain transcription factors [18]. These regulatory pathways ensure proper formation of xylem and phloem, necessary for long-distance transport of assimilates and signaling molecules.

Leaves, as the primary photosynthetic organs, display a distinct transcriptomic landscape marked by genes for chloroplast development, light response, and carbon fixation (e.g., Rubisco subunits, chlorophyll a/b binding proteins) [2]. Comparative analyses between photosynthetically active leaves and non-photosynthetic tissues reveal tightly regulated metabolic compartmentalization and tissue-specific expression of plastid-related genes. Flower buds, meanwhile, initiate a reproductive gene program, including MADS-box transcription factors and floral organ identity genes, such as APETALA and AGAMOUS homologs [22]. This ensures proper floral organ specification and timing of reproductive transitions.

Despite these advances, our understanding of how gene expression dynamics differ across a broad tissue set—from seed developmental stages through vegetative tissues to early seedling—is still incomplete in soybean. Moreover, integrative approaches combining principal component analysis (PCA), differential expression (DE), and co-expression network modeling in the same dataset remain less common.

The objective of this study was to perform an integrative re-analysis of soybean transcriptomic data across developmental and vegetative tissues to identify coordinated gene expression patterns, differentially expressed genes, and co-expression relationships underlying tissue specialization. Unlike previous studies that focused on individual aspects of soybean transcriptomics, this study integrates multiple analytical layers—including variance-based gene selection, differential expression, correlation structure, and network analysis—to identify coordinated transcriptional programs underlying tissue specialization.

## 2. Results

The dataset analyzed (GSE29163) comprises RNA-seq expression profiles across 12 soybean tissues. The dataset does not include biological replicates for each tissue, and each sample represents a distinct developmental stage or organ. Therefore, downstream statistical analyses should be interpreted as exploratory rather than confirmatory.

### 2.1. Composition of the Soybean Transcriptomic Dataset

The transcriptomic dataset analyzed in this study comprised samples representing a broad range of soybean developmental stages and tissue types. In total, twelve tissues were included, spanning multiple seed developmental stages as well as vegetative and reproductive organs. Seed tissues included globular, heart, cotyledon, embryo, dry seed, mid-mature seed, and late-mature seed stages, whereas non-seed tissues consisted of leaf, root, stem, flower bud, and seedling 6 DAI.

A summary of the dataset composition is shown in Supplementary Figure S1, which illustrates the relative number of samples representing seed versus vegetative or reproductive tissues. Seed tissues accounted for seven of the twelve samples, reflecting the emphasis on multiple stages of embryo and seed maturation. Vegetative and reproductive tissues accounted for the remaining five samples. This dataset structure enabled comparative analysis of transcriptional programs associated with seed development relative to those operating in vegetative organs.

### 2.2. Principal Component Analysis Reveals Clear Separation of Tissue Types

**Figure 1:**
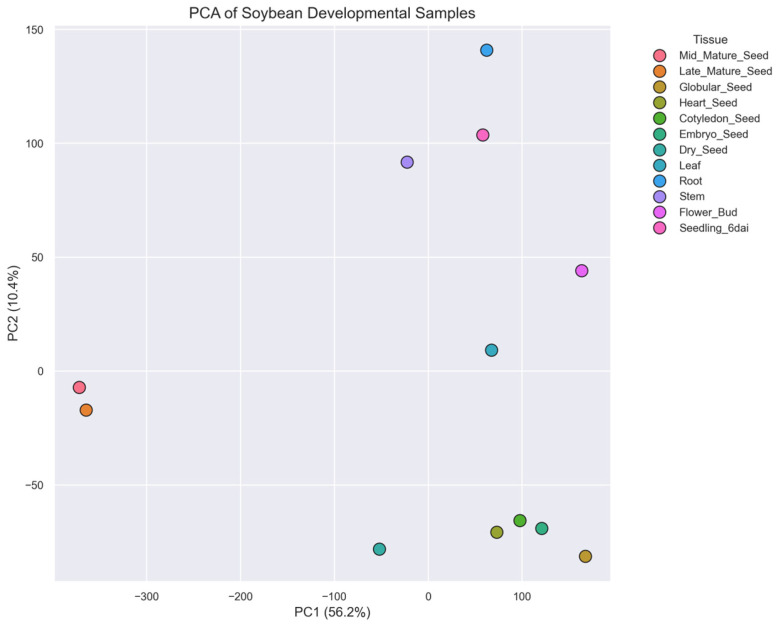
Principal component analysis of soybean tissues. Scatter plot of the first two principal components derived from the log_2_-transformed expression matrix, showing global transcriptional relationships among seed developmental stages and vegetative tissues.

PCA was performed using the full set of filtered genes (after removal of genes with >50% missing values and log_2_ transformation). PCA of the log_2_-transformed soybean expression data revealed a clear separation of tissue types along the first two principal components (Figure 1). Together, these components explained 66.6% of the total variance, with PC1 accounting for 56.2% and PC2 explaining 10.4% of the variance.

Seed-related tissues (Mid_Mature_Seed, Late_Mature_Seed, Globular_Seed, Heart_Seed, Cotyledon_Seed, Embryo_Seed, and Dry_Seed) clustered together and were clearly separated from vegetative organs (Leaf, Root, Stem) and reproductive tissues (Flower_Bud and Seedling_6dai). The strong separation along PC1 indicates that the primary axis of transcriptomic variation corresponds to the developmental distinction between seed and non-seed tissues.

Within the seed cluster, earlier embryonic stages such as globular and heart stages formed closely related groups, whereas mature seed tissues occupied distinct positions along the PCA axes. These patterns suggest that progressive developmental transitions during seed maturation are accompanied by substantial transcriptional remodeling. Conversely, vegetative tissues formed a separate cluster reflecting shared physiological functions such as photosynthesis, nutrient transport, and structural growth.

### 2.3. Highly Variable Genes Distinguish Developmental and Vegetative Programs

**Figure 2:**
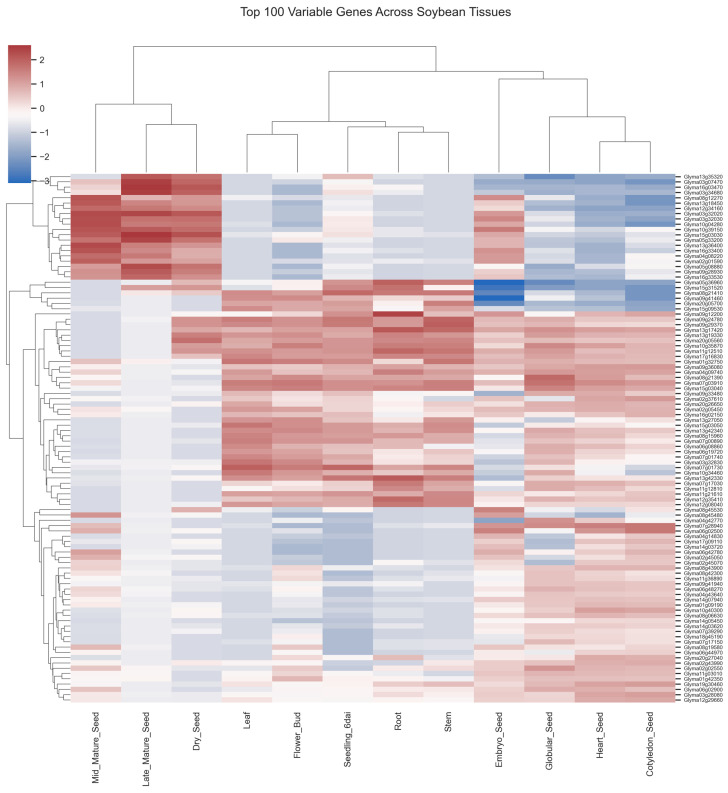
Heatmap of the top 100 most variable genes across soybean tissues. Hierarchically clustered heatmap showing row-wise standardized expression (z-score) of the 100 genes with the highest variance across samples, highlighting distinct transcriptional programs associated with seed and vegetative tissues.

Highly variable genes were defined as those with the highest variance across all samples. Specifically, the top 100 genes ranked by variance across samples were selected for downstream clustering analysis (Figure 2).

The resulting heatmap revealed clear gene expression patterns distinguishing seed and non-seed tissues. Two major gene clusters were apparent. One cluster showed elevated expression across multiple seed developmental stages but reduced expression in vegetative tissues, suggesting roles in seed-specific processes such as embryo development, storage compound accumulation, and desiccation tolerance. The second cluster exhibited higher expression in vegetative organs, consistent with genes involved in photosynthesis, structural growth, and metabolic activity in actively growing tissues.

Clustering of the samples further supported the separation observed in the PCA. Seed developmental stages grouped together, whereas vegetative organs and reproductive tissues formed separate clusters. These patterns highlight the presence of distinct transcriptional programs associated with seed maturation versus vegetative growth.

### 2.4. Differential Gene Expression Between Seed and Non-Seed Tissues

Differential gene expression analysis was performed to identify genes exhibiting coordinated transcriptional differences between seed and non-seed tissues. Statistical testing identified 9785 genes that satisfied the thresholds of FDR < 0.05 and |log_2_FC| > 1.

**Figure 3:**
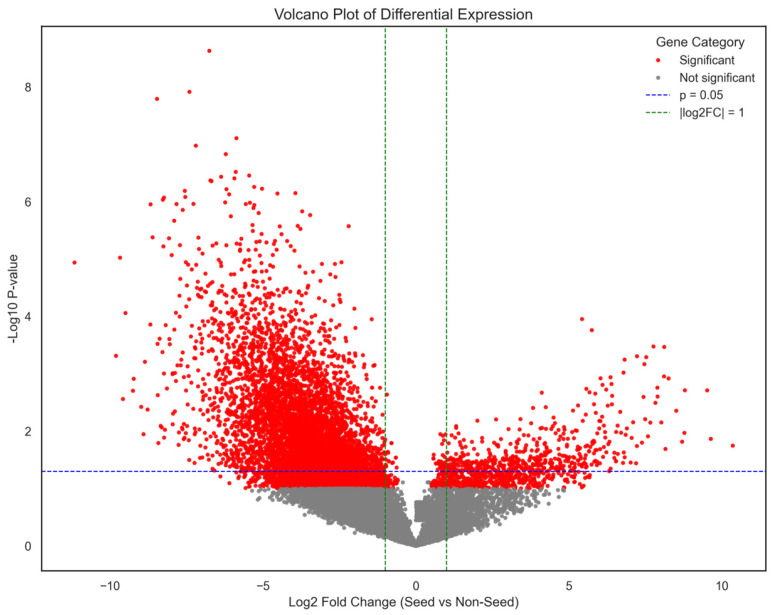
Volcano plot of differential gene expression between seed and non-seed tissues. Genes are plotted based on log_2_ fold change and −log_10_(*p*-value), with significantly differentially expressed genes highlighted in red. The horizontal dashed blue line indicates the significance threshold (*p* = 0.05), while the vertical dashed green lines represent log_2_ fold-change thresholds (±1), illustrating the magnitude and direction of transcriptional changes.

Among these, 1139 genes were significantly upregulated in seed tissues, whereas 8646 genes were downregulated relative to vegetative and reproductive organs. The results are visualized using a volcano plot (Figure 3), which illustrates the distribution of genes according to fold change and statistical significance.

The volcano plot reveals a strong asymmetry in differential expression patterns. A large proportion of genes exhibited lower expression in seed tissues relative to vegetative organs, reflecting the suppression of many growth-associated processes during seed maturation. In contrast, a smaller but distinct set of genes showed strong upregulation in seed tissues, likely representing genes involved in embryo development, storage reserve synthesis, and seed maturation pathways.

### 2.5. Expression Patterns of the Top Differentially Expressed Genes

**Figure 4:**
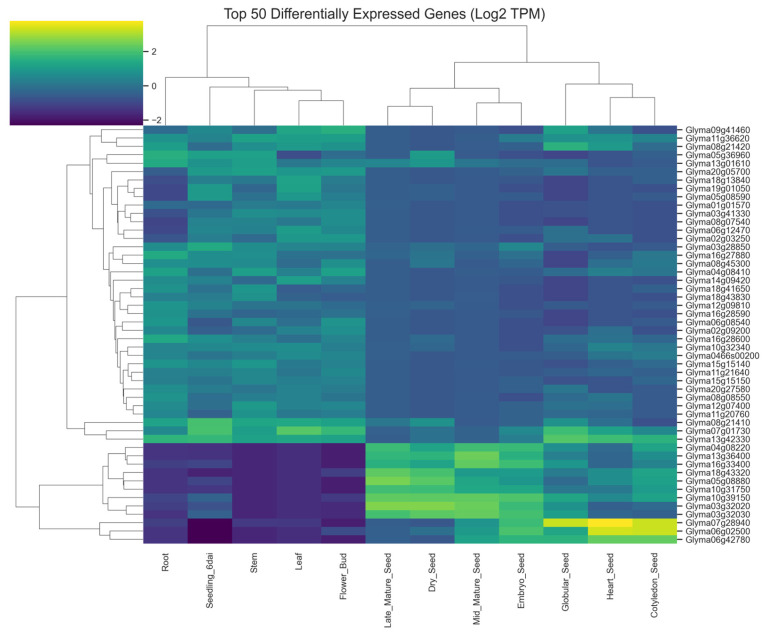
Heatmap of the top 50 differentially expressed genes. Clustered heatmap displaying log_2_-transformed expression values of genes with the largest absolute fold changes between seed and non-seed tissues, revealing strong tissue-specific expression patterns.

To further explore transcriptional differences between tissues, the 50 genes with the largest absolute log_2_ fold changes were selected and visualized using hierarchical clustering (Figure 4). The heatmap revealed pronounced differences in expression patterns between seed and vegetative tissues.

Genes highly expressed in seed tissues formed distinct clusters corresponding to developmental stages of embryo formation and seed maturation. Many of these genes are consistent with known seed-related functional categories, including proteins associated with lipid storage, seed storage proteins, and stress-related protective proteins such as late embryogenesis abundant (LEA) proteins.

Conversely, genes highly expressed in vegetative tissues showed elevated expression in leaf, root, and stem samples. These genes are likely associated with photosynthetic processes, cell wall biosynthesis, and general metabolic functions required for vegetative growth. Together, these patterns demonstrate strong tissue-specific transcriptional specialization across soybean developmental stages.

### 2.6. Functional Characterization of Seed-Upregulated Genes

**Figure 5:**
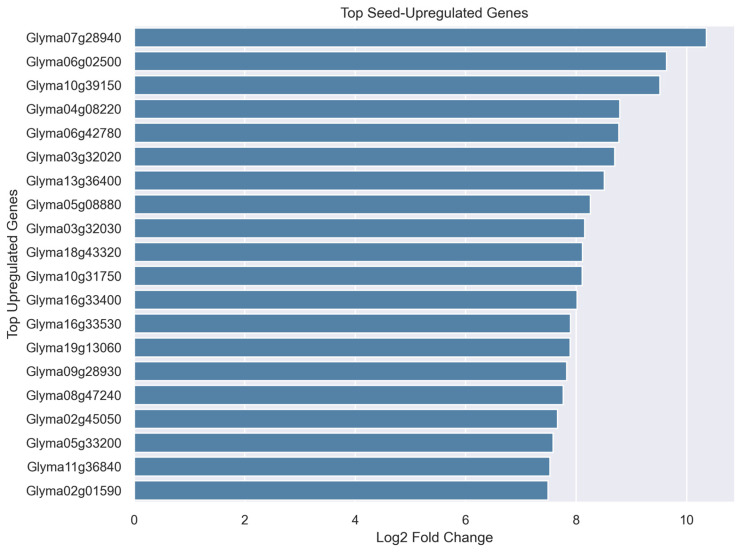
Top seed-upregulated genes ranked by log_2_ fold change. Bar plot showing the genes with the highest positive log_2_ fold change in seed tissues relative to non-seed tissues. Genes are ordered by decreasing fold change, highlighting candidate genes associated with seed development, including stress-responsive and membrane-associated proteins.

To gain functional insight into genes preferentially expressed in seed tissues, we examined the top upregulated genes identified in the differential expression analysis (Figure 5) and integrated available functional annotations from SoyBase. Among these, several genes are associated with stress response and cellular adaptation processes that are characteristic of seed maturation.

Notably, Glyma06g02500 is annotated as an HVA22-like protein G (IPR004345; TB2/DP1/HVA22-related protein), a protein family known to be involved in abscisic acid (ABA)-mediated stress responses and vesicle trafficking under dehydration conditions. HVA22-like proteins have been implicated in protecting cellular integrity during water deficit and are often associated with late stages of seed development, when desiccation tolerance is acquired.

In addition, Glyma04g082200 encodes a protein containing a stress-responsive A/B barrel domain (IPR011008), which is commonly associated with proteins involved in environmental stress adaptation and metabolic regulation. The presence of this domain further supports the activation of stress-responsive pathways in seed tissues. Another gene, Glyma13g364000, is currently annotated as a protein of unknown function; however, its localization to the plasma membrane and broad expression across multiple plant structures and developmental stages suggest a potential role in membrane-associated processes during seed maturation.

Although functional annotations are limited for several of the top upregulated genes, the available evidence points toward enrichment of pathways related to stress response, cellular protection, and membrane dynamics. These processes are well recognized as essential components of seed maturation, particularly in the acquisition of desiccation tolerance and long-term viability [23,24]. The observed expression patterns are also consistent with established Gene Ontology classifications describing seed development and stress adaptation processes [25].

Collectively, these findings indicate that seed-upregulated genes reflect coordinated transcriptional programs associated with stress resilience, cellular protection, and developmental specialization. Furthermore, the identification of both annotated and uncharacterized genes highlights opportunities for future functional studies aimed at elucidating novel regulators of soybean seed development.

### 2.7. Co-Expression Network Reveals Distinct Tissue Modules

**Figure 6:**
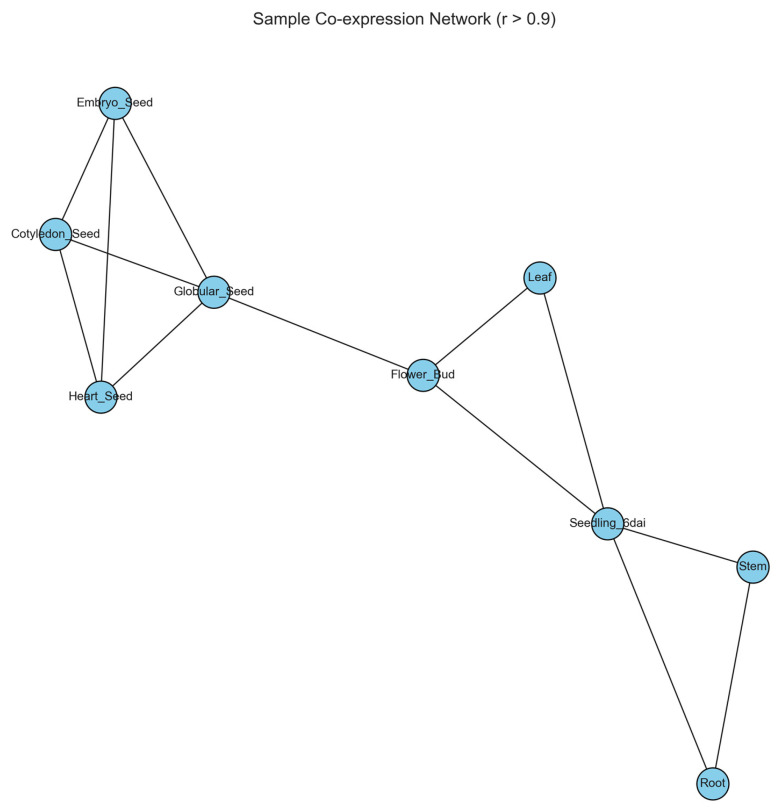
Sample co-expression network of soybean tissues. Network representation of transcriptional similarity among tissues based on Pearson correlation coefficients (|r| > 0.9), with nodes representing tissues and edges indicating strong correlations.

To explore transcriptional relationships among tissues, a co-expression network was constructed using Pearson correlation coefficients greater than 0.9. The resulting network structure is shown in Figure 6. In this network, nodes represent tissues and edges represent strong correlations in gene expression profiles. Seed developmental stages formed a tightly interconnected module, reflecting their highly similar transcriptional programs. Early embryonic stages, including globular, heart, cotyledon, and embryo tissues, exhibited particularly strong connectivity within the network.

Vegetative and reproductive tissues formed a separate module consisting of leaf, root, stem, flower bud, and seedling samples. Connections among these tissues indicate shared transcriptional activity associated with vegetative growth and organ development. The network structure therefore reflects the biological organization of soybean tissues into functionally related transcriptional modules. The co-expression network includes only tissues connected by strong correlations (|r| > 0.9). As a result, only 9 tissues are represented as nodes, since some samples did not meet this threshold.

### 2.8. Global Transcriptomic Similarity Among Soybean Tissues

**Figure 7:**
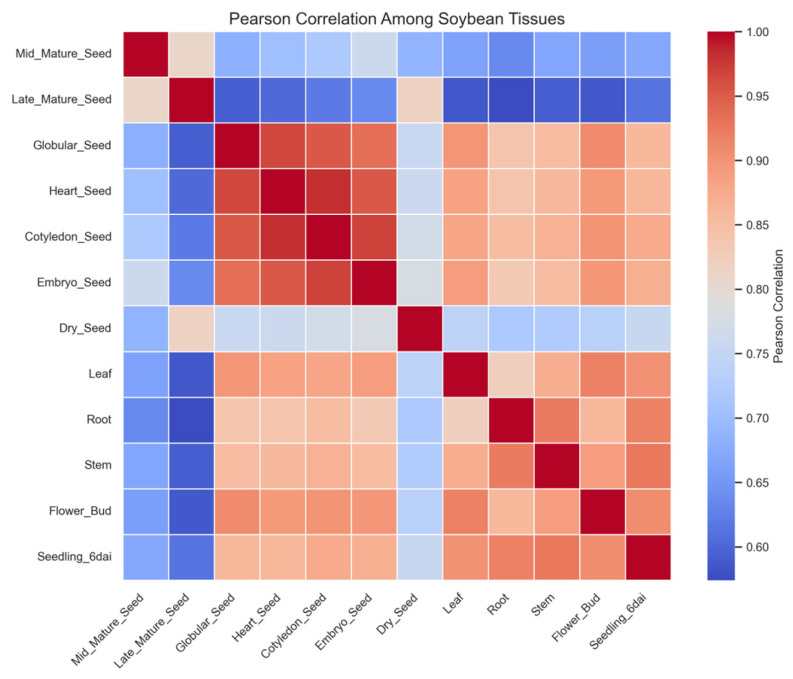
Pearson correlation matrix of soybean tissue transcriptomes. Heatmap showing pairwise Pearson correlation coefficients among all samples using log_2_-transformed gene expression values, illustrating global transcriptional similarity across tissues.

Pairwise Pearson correlation coefficients were calculated to quantify the similarity of transcriptomes across all tissues. The resulting correlation matrix is shown as a heatmap in Figure 7. High correlation values were observed among seed developmental stages, indicating that these tissues share closely related transcriptional programs. In particular, globular, heart, cotyledon, and embryo tissues displayed strong pairwise correlations, consistent with their sequential developmental progression during embryogenesis.

Vegetative tissues also exhibited strong correlations among themselves, particularly between leaf, stem, and root samples. In contrast, correlations between seed and vegetative tissues were generally lower, reflecting substantial divergence in transcriptional programs between developmental programs associated with embryo maturation and vegetative growth.

### 2.9. Distribution of Differential Expression Magnitudes

**Figure 8:**
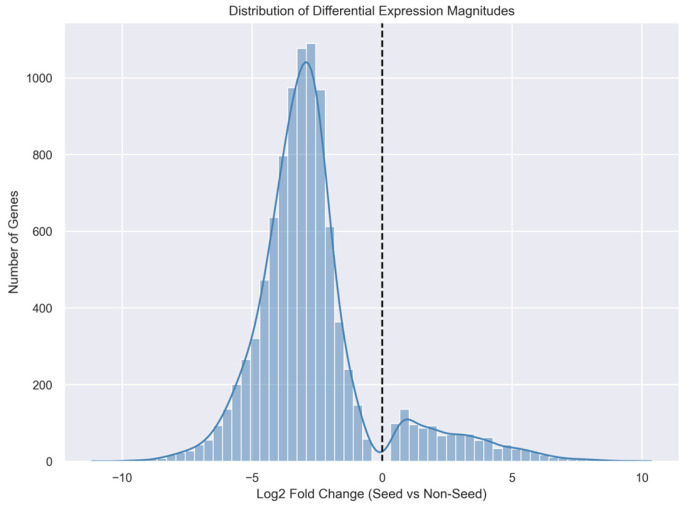
Distribution of differential gene expression magnitudes between seed and non-seed tissues. Histogram showing the distribution of log_2_ fold-change values across all genes, with a kernel density curve (blue line) illustrating the overall distribution. The vertical dashed black line indicates no change in expression (log_2_ fold change = 0), highlighting the extent and direction of transcriptional differences.

Figure 8 shows the distribution of log_2_ fold-change values across all genes, reflecting the global magnitude and direction of transcriptional differences. Most genes exhibited fold changes near zero, indicating similar expression levels between seed and non-seed tissues. However, the distribution displayed extended tails representing genes with large positive or negative fold changes.

Genes with large positive fold changes correspond to those strongly upregulated in seed tissues, whereas genes with large negative fold changes are preferentially expressed in vegetative organs. This distribution highlights the coexistence of seed-specific transcriptional activation and repression of vegetative gene programs.

### 2.10. Global Summary of Differentially Expressed Genes

A summary of the number of genes significantly upregulated or downregulated in seed tissues is shown in Supplementary Figure S2. Differential expression analysis identified 1139 genes upregulated in seed tissues and 8646 genes downregulated relative to non-seed tissues.

The predominance of downregulated genes supports the fact that many metabolic and physiological pathways active in vegetative tissues are suppressed during seed development. This observation is consistent with the transition from actively growing tissues to specialized reproductive structures focused on storage compound accumulation and embryo maturation.

### 2.11. MA Plot of Gene Expression Changes

**Figure 9:**
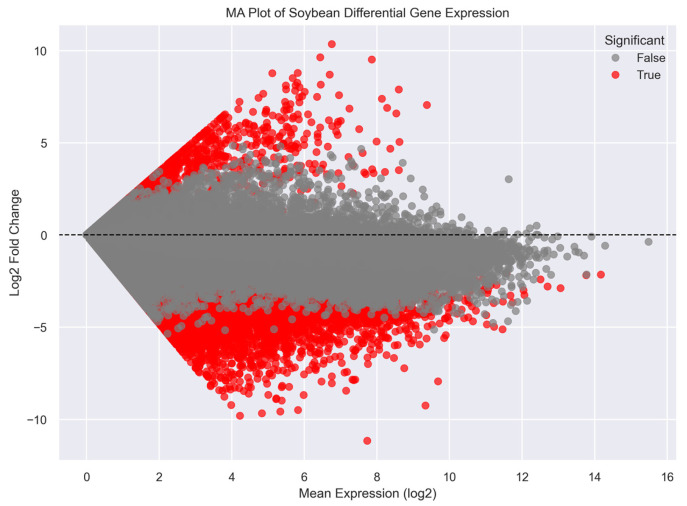
MA plot of soybean differential gene expression. Scatter plot showing the relationship between mean gene expression and log_2_ fold change across all genes, illustrating expression-dependent transcriptional variation.

To further evaluate differential expression patterns, an MA plot was constructed showing the relationship between mean gene expression levels and log_2_ fold changes (Figure 9). Most genes clustered around the horizontal axis, indicating relatively stable expression between the two tissue groups. However, a substantial number of genes displayed large positive or negative fold changes across a range of expression levels. Highly significant genes were distributed across both high and moderate expression ranges, suggesting that transcriptional changes associated with seed development involve genes with diverse expression intensities.

### 2.12. Hierarchical Clustering Confirms Tissue Relationships

**Figure 10:**
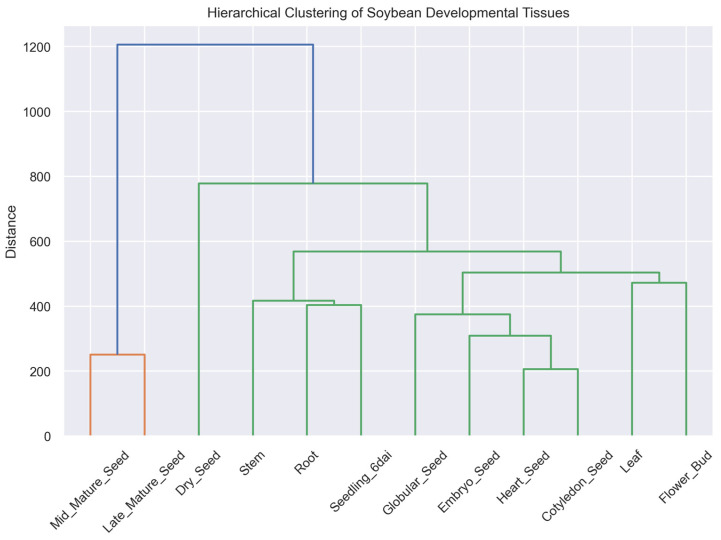
Hierarchical clustering of soybean developmental tissues based on gene expression profiles. Dendrogram showing relationships among tissues using hierarchical clustering of log_2_-transformed expression data. The y-axis represents clustering distance. Seed tissues cluster separately from vegetative and reproductive tissues, reflecting distinct transcriptional programs. Branch colors are assigned automatically by the clustering algorithm and do not represent predefined biological groups.

Hierarchical clustering analysis was performed to further examine relationships among soybean tissues based on their global transcriptomic profiles. The resulting dendrogram (Figure 10) revealed clear grouping of tissues according to developmental and physiological characteristics.

Seed tissues clustered together, with closely related embryonic stages forming subclusters within the broader seed group. Vegetative tissues formed a separate branch of the dendrogram, reflecting their shared transcriptional programs associated with growth and metabolic activity.

Notably, reproductive tissues such as flower buds and early seedlings showed intermediate relationships, linking vegetative and developmental clusters. Overall, the clustering results reinforce the patterns observed in PCA, correlation analysis, and network analysis, confirming that soybean tissues exhibit strong transcriptional organization corresponding to their developmental roles. Hierarchical clustering revealed groupings consistent with PCA results but also highlighted finer relationships among tissues, particularly within seed developmental stages, suggesting subtle transcriptional differences not fully captured by PCA.

### 2.13. Identification of Candidate Genes Associated with Seed Development

The top differentially expressed genes identified in this study represent candidate genes associated with soybean seed development and tissue specialization. Notably, several genes with strong upregulation in seed tissues are associated with storage protein accumulation, lipid metabolism, and stress tolerance. These include genes encoding late embryogenesis abundant (LEA) proteins, oleosins, and other seed-specific proteins. Conversely, genes downregulated in seed tissues are primarily associated with photosynthesis, cell wall biosynthesis, and vegetative growth.

To further highlight candidate genes associated with seed development, the top upregulated genes ranked by log_2_ fold change were visualized (Figure 10). These genes exhibited strong preferential expression in seed tissues compared to vegetative organs. Several of the highly upregulated genes are likely associated with seed-specific biological processes, including storage protein accumulation, lipid metabolism, and stress protection mechanisms. The identification of these candidate genes provides a focused set of targets for future functional validation and crop improvement studies.

## 3. Discussion

The integrative transcriptomic analysis presented here provides a comprehensive view of gene expression dynamics across soybean developmental and vegetative tissues. By combining multivariate statistical approaches, differential expression analysis, and network-based methods, this study reveals key transcriptional features associated with soybean organ specialization and seed development.

One of the most prominent observations from this study is that tissue identity represents the dominant axis of transcriptional variation in soybean. Principal component analysis revealed strongly coordinated gene expression programs, with the first two principal components explaining more than two-thirds of the total variance in the dataset. This separation reflects large-scale transcriptional reprogramming associated with the transition from vegetative growth to reproductive development. Similar patterns have been reported in previous soybean transcriptome atlases and developmental profiling studies, where seed maturation stages cluster separately from leaf, root, and stem tissues [2,16,22,26]. The distinct clustering of embryonic stages further is consistent with the fact that progressive developmental transitions during seed maturation are accompanied by large-scale shifts in transcriptional activity [27].

The analysis of the top 100 most variable genes further highlights the existence of distinct regulatory programs associated with seed and vegetative tissues. Hierarchical clustering of these genes revealed two major expression modules: one predominantly expressed in seed developmental stages and another enriched in vegetative organs. Such patterns are consistent with previous transcriptomic atlases showing that plant organs maintain specialized transcriptional networks adapted to their physiological roles [1,5,18]. In vegetative tissues, highly expressed genes are often associated with processes such as photosynthesis, carbon metabolism, and structural growth. In contrast, genes highly expressed in seeds are typically linked to embryo development, storage compound synthesis, and stress tolerance mechanisms required for seed maturation.

Differential gene expression analysis further demonstrated the magnitude of transcriptional reprogramming that occurs between seed and non-seed tissues. In total, nearly ten thousand genes were significantly differentially expressed, including more than eight thousand genes downregulated in seeds relative to vegetative tissues. The predominance of downregulated genes suggests that many metabolic pathways active in vegetative tissues become suppressed during seed development. Such repression is expected during the transition from actively growing organs toward specialized reproductive structures focused on storage reserve accumulation and embryo maturation. Similar large-scale transcriptional shifts have been observed in other studies of soybean seed development and legume embryogenesis [10,11,22,26,28].

The examination of the top differentially expressed genes revealed several functional categories characteristic of seed maturation processes. Many of the genes highly expressed in seeds encode proteins involved in storage compound accumulation, lipid body formation, and stress protection. These include genes encoding storage proteins, oleosin family proteins associated with lipid droplets, and late embryogenesis abundant (LEA) proteins. LEA proteins and related stress-response proteins are known to play critical roles in protecting cellular structures during dehydration and seed desiccation [2,29,30]. Their elevated expression during seed maturation reflects the physiological requirement to prepare the embryo for dormancy and long-term survival.

The analysis also revealed that dry seeds exhibit a highly specialized transcriptional state. Previous studies have shown that dry seeds accumulate transcripts encoding dehydrins, small heat shock proteins, and other stress-protective molecules during late maturation stages [2,17,31,32]. These proteins contribute to membrane stabilization, protein protection, and tolerance to cellular dehydration during seed desiccation. The transcriptomic patterns observed in this study are consistent with these findings, as dry and late-mature seeds occupy distinct positions in the PCA and clustering analyses relative to earlier embryonic stages and vegetative tissues.

The identification of top upregulated genes provides additional biological insight beyond global expression patterns. These genes represent strong candidate regulators or effectors of seed development and maturation. Many are likely involved in processes such as storage reserve accumulation, lipid body formation, and desiccation tolerance, which are essential for seed viability. Highlighting these candidate genes addresses an important gap in transcriptomic studies by linking global expression patterns to specific molecular targets that can be explored in functional genomics and breeding applications.

Network-based approaches provided additional insight into the organization of transcriptional programs across soybean tissues. The co-expression network constructed from highly correlated samples revealed strong connectivity among seed developmental stages, indicating that these tissues share highly coordinated gene expression patterns. Such tight clustering likely reflects the presence of common regulatory hubs controlling developmental pathways during embryo formation and maturation. Similar network-based analyses in soybean have demonstrated that gene expression during seed development is governed by highly interconnected regulatory modules [33,34]. In contrast, vegetative tissues exhibited slightly more modular network organization, reflecting the diversity of physiological processes operating across organs such as leaves, roots, and stems.

Correlation analysis and hierarchical clustering further supported these observations. Both approaches consistently grouped seed tissues together while separating them from vegetative organs. The concordance among multiple analytical methods—including PCA, clustering, correlation analysis, and network modeling—provides strong evidence that soybean tissues exhibit highly structured transcriptomic organization corresponding to their biological roles.

Beyond seed development, the transcriptional patterns observed in vegetative tissues reflect dynamic transcriptional responses to environmental and developmental signals [35]. Leaf tissues showed expression patterns associated with photosynthetic metabolism, whereas root and stem tissues exhibited transcriptional profiles linked to nutrient uptake, structural growth, and transport processes. Such divergence among organ-specific transcriptomes reflects the broader principle of transcriptional compartmentalization that underlies plant development. These findings are consistent with emerging pan-tissue transcriptomic maps demonstrating that plant organs maintain distinct gene expression signatures tailored to their physiological functions [18,36,37].

From an applied perspective, the gene sets identified in this study may provide valuable candidates for improving soybean crop traits. Genes strongly upregulated in seeds are particularly attractive targets for breeding programs aiming to enhance seed composition, including oil and protein content [15,38]. Likewise, genes associated with stress tolerance and desiccation protection could contribute to improved seed vigor and storage longevity. The high degree of transcriptional coordination observed among seed developmental stages also suggests that a limited number of representative stages may capture much of the transcriptomic variability associated with seed maturation, potentially enabling more efficient experimental designs in future studies.

Despite these insights, several limitations should be acknowledged. Because the analysis relies on publicly available datasets, variation in experimental conditions such as sequencing platforms, library preparation methods, and sample processing may introduce technical heterogeneity. In addition, the differential expression analysis employed simplified thresholds that do not account for all potential confounding variables, including genotype differences or batch effects. Future studies should address these limitations by incorporating biological replicates, multi-genotype datasets, and more robust statistical frameworks for RNA-seq analysis.

Further work will be required to validate candidate genes identified in this study, which may serve as targets for crop improvement and trait optimization [39]. Functional characterization using reverse genetics approaches, such as CRISPR/Cas-mediated gene editing or transgenic expression studies, will be essential to determine the precise biological roles of these genes in soybean development. Integration of transcriptomic data with other omics layers—including proteomics, metabolomics, and epigenomic profiling—may also provide a more comprehensive understanding of the regulatory mechanisms controlling seed maturation.

Another limitation of this study is the aggregation of multiple seed developmental stages into a single group for differential expression analysis, which may obscure stage-specific transcriptional dynamics. Future analyses using pairwise or time-series approaches could provide higher-resolution insights into developmental regulation. The absence of biological replicates limits statistical robustness. In addition, incorporating gene family-level clustering or pathway-based analysis may further refine the interpretation of tissue-specific transcriptional programs.

In conclusion, this study integrates multivariate analysis, variance-based gene filtering, differential expression profiling, and network modeling to provide a comprehensive overview of transcriptional dynamics across soybean developmental tissues. The results highlight the strong transcriptional specialization that distinguishes seed developmental stages from vegetative organs and identify candidate genes associated with key biological processes such as embryo maturation, nutrient storage, and desiccation tolerance. These findings contribute to a growing body of transcriptomic resources that enhance our understanding of soybean development and provide a foundation for future functional genomics and crop improvement efforts.

Compared to previous soybean transcriptome atlases, this study emphasizes the coordinated structure of gene expression across tissues rather than focusing solely on individual gene differences. The integration of multiple analytical layers provides a broader systems-level understanding of soybean development.

From an applied perspective, the identified gene sets may serve as targets for improving seed composition, stress tolerance, and developmental efficiency through breeding or genetic engineering.

Beyond general functional categories, the enrichment of genes associated with nutrient reservoir activity and desiccation tolerance indicates coordinated regulation by seed-specific transcriptional networks. Previous studies have identified key regulators such as LEAFY COTYLEDON (LEC), ABSCISIC ACID INSENSITIVE (ABI), and WRINKLED1 (WRI1) as central drivers of seed maturation and storage compound accumulation. The enrichment patterns observed in this study are consistent with activation of these regulatory modules, suggesting that the identified gene sets may represent downstream targets of conserved seed developmental regulators.

## 4. Methods

### 4.1. Data Acquisition and Preprocessing

Transcriptomic datasets were obtained from the Gene Expression Omnibus (GEO) under accession GSE29163 [2], which contains RNA-seq expression profiles from multiple soybean (*Glycine max*) tissues representing both developmental and vegetative stages. Samples included seed developmental stages (globular, heart, cotyledon, embryo, dry seed, mid-mature seed, and late-mature seed), vegetative tissues (leaf, root, and stem), a reproductive organ (flower bud), and early post-germination tissue (seedling 6 DAI). No biological replicates are available.

Raw expression files were downloaded and merged into a unified gene expression matrix following commonly recommended RNA-seq data processing practices [40]. Gene identifiers were standardized across files, and sample names were simplified for readability during downstream analysis. Genes with missing values in more than 50% of samples were removed. Remaining missing values were replaced with zero as a pragmatic approach to maintain matrix completeness; however, this may underestimate low-expression genes and should be interpreted cautiously.

Expression values were transformed using the function log_2_(x + 1) to stabilize variance and reduce the influence of highly expressed genes. After filtering, the final dataset consisted of approximately 74,818 genes across 12 tissue samples [2]. Samples were grouped into two biological categories for comparative analysis: seed tissues (all developmental seed stages) and non-seed tissues (vegetative and reproductive organs).

### 4.2. Overview of Tissue Composition

To illustrate the distribution of biological samples included in the study, tissues were categorized into seed developmental stages and vegetative/reproductive tissues. The number of samples representing each category was summarized to provide an overview of the dataset composition.

### 4.3. Principal Component Analysis (PCA)

PCA was performed on the filtered and log_2_-transformed gene expression data to evaluate global transcriptional relationships among samples. Prior to PCA, gene expression values were standardized using the StandardScaler implementation in the Scikit-learn library [41]. Dimensionality reduction was then performed using PCA, and the first two principal components were extracted to visualize the primary axes of transcriptional variation among soybean tissues.

### 4.4. Identification of Highly Variable Genes

To identify genes exhibiting the greatest transcriptional variability across tissues, gene-wise variance was calculated across the entire log_2_-transformed dataset. The top 100 genes with the highest variance were selected for clustering analysis. Expression values for these genes were standardized using row-wise z-score normalization to emphasize relative expression patterns across tissues. Hierarchical clustering was performed using Euclidean distance and average linkage to identify groups of genes with similar expression patterns across developmental and vegetative samples.

### 4.5. Differential Gene Expression Analysis

Differential gene expression analysis was conducted to identify genes whose expression showed functional specialization, following commonly used statistical frameworks for RNA-seq differential expression analysis [42,43]. For each gene, the log_2_ fold change (log_2_FC) was calculated as the difference between the mean expression of seed tissues and the mean expression of non-seed tissues. Statistical significance was evaluated using Welch’s two-sample *t*-test, which accounts for unequal variances between groups. *p*-values were adjusted for multiple hypothesis testing using the Benjamini–Hochberg false discovery rate (FDR) correction. The top differentially expressed genes were defined as those with the largest absolute log_2_ fold change among genes meeting the significance threshold (FDR < 0.05). The distribution of gene expression changes was calculated using log_2_ fold-change values for all genes between seed and non-seed tissues. Using these thresholds, a total of 9785 genes were identified as differentially expressed, including 1139 genes up-regulated in seed tissues and 8646 genes down-regulated relative to non-seed tissues.

This grouping strategy was designed to capture global transcriptional differences between seed and non-seed tissues. However, it does not account for stage-specific or tissue-specific variation, which represents an important direction for future analyses.

Due to the absence of biological replicates in the dataset, standard RNA-seq differential expression tools such as DESeq2 or edgeR could not be reliably applied. Therefore, Welch’s *t*-test was used as an exploratory statistical approach to identify broad transcriptional differences between groups. The results should be interpreted as indicative of global expression trends rather than definitive statistical inference.

### 4.6. Visualization of Differential Expression Patterns

Several visualization approaches were used to examine the distribution and magnitude of differential expression results. Volcano plots were generated to display the relationship between log_2_ fold change and statistical significance. Histograms of log_2_ fold-change values were constructed to visualize the overall distribution of differential expression magnitudes across genes. An MA plot was also produced to examine the relationship between mean expression levels and log_2_ fold changes across genes. In addition, the number of up-regulated and down-regulated genes was summarized to provide an overview of developmental programming.

### 4.7. Heatmap of Differentially Expressed Genes

To examine transcriptional patterns among the most strongly regulated genes, the top 50 genes with the largest absolute log_2_ fold changes were selected. Expression values for these genes were visualized using a clustered heatmap based on log_2_-transformed expression values. Hierarchical clustering was applied to both genes and samples using Euclidean distance and average linkage to reveal groups of genes with similar expression patterns across tissues.

### 4.8. Correlation Analysis of Tissue Transcriptomes

To evaluate global transcriptional similarity among tissues, a Pearson correlation matrix was calculated using the log_2_-transformed expression dataset. The resulting matrix was visualized as a heatmap in which correlation coefficients reflected the overall similarity between pairs of tissues. This analysis allowed identification of closely related transcriptional programs across developmental stages and vegetative organs.

### 4.9. Hierarchical Clustering of Tissue Transcriptomes

Hierarchical clustering was performed to assess global similarity among soybean tissues based on transcriptomic profiles. Pairwise distances between samples were calculated using Euclidean distance applied to the log_2_-transformed expression matrix. Agglomerative hierarchical clustering was then performed using the average linkage method, which iteratively groups samples based on their pairwise similarity. The resulting dendrogram illustrates relationships among tissues and highlights clusters corresponding to seed developmental stages and vegetative organs. Clustering was implemented using functions from the SciPy v1.13 hierarchical clustering module [44] and visualized using Matplotlib v3.10.

### 4.10. Co-Expression Network Analysis

To further explore transcriptional relationships among tissues, a co-expression network was constructed based on Pearson correlation coefficients, an approach commonly used in gene co-expression network analysis [45,46]. Pairwise correlations were calculated between samples, and edges were drawn between nodes representing tissues when the correlation coefficient exceeded r > 0.9. The resulting network was modeled as an undirected graph using the NetworkX Python library [45]. Node positions were determined using a spring-layout algorithm to optimize visualization of highly connected sample groups.

### 4.11. Software and Computational Environment

All analyses were performed using Python v3.10 [47] within a Jupyter Notebook v7.4 environment [48]. Numerical computations and matrix operations were conducted using NumPy v1.26 [49], while Pandas v2.2 [50] was used for data manipulation and table handling. Data visualization was performed using Matplotlib 3.10 [51] and Seaborn v0.13 [52], which enabled the generation of high-resolution publication-quality figures. Hierarchical clustering and principal component analysis were implemented using Scikit-learn v1.7 [41], and network analysis was performed using NetworkX 3.4 [45].

## 5. Conclusions

In this study, we performed an integrative transcriptomic analysis of soybean developmental and vegetative tissues to characterize global patterns of gene expression. Multivariate analyses demonstrated that tissue identity—particularly the distinction between seed and non-seed tissues—represents the dominant source of transcriptional variation.

Analysis of highly variable and differentially expressed genes revealed distinct transcriptional programs associated with seed maturation and vegetative growth. Network and correlation analyses further demonstrated strong transcriptional coherence among seed tissues, indicating coordinated regulatory processes underlying embryo development and storage accumulation.

Although limited by the absence of biological replicates, this study provides a systems-level framework for understanding transcriptional organization across soybean tissues. The identified gene sets represent valuable candidates for future functional genomics studies and breeding strategies aimed at improving seed composition, yield, and stress resilience.

## Data Availability

The transcriptomic dataset analyzed in this study is publicly available from the NCBI Gene Expression Omnibus (GEO) under accession number GSE29163. The processed expression matrices, differential expression results, and the complete Jupyter Notebook used for data preprocessing, statistical analysis, and figure generation are publicly available at the corresponding author’s GitHub repository: https://github.com/abdelmajidk/Transcriptomic-Profiling-Soybean (accessed on 22 March 2026).

## Supplementary Materials

The following supporting information can be downloaded at https://www.mdpi.com/article/10.3390/plants15071002/s1, Figure S1. Composition of soybean transcriptomic dataset. Bar plot showing the number of samples representing seed developmental stages and vegetative or reproductive tissues included in the analysis. Figure S2. Summary of differentially expressed genes. Bar plot showing the number of genes significantly upregulated and downregulated in seed tissues compared to non-seed tissues based on FDR and fold-change thresholds.
