# Supplementary material for: Integrative Transcriptomic Analysis and Co-Expression Network Characterization of Soybean Developmental Tissues
Source: Plants (Basel). 2026 Mar 25;15(7):1002. doi: 10.3390/plants15071002 (PMC13075193; doi:10.3390/plants15071002)
Supplement: Supplementary file 1 [file plants-15-01002-s001.zip › plants-4207788-supplementary.pdf]

Supplementary Materials
Supplementary Figures:

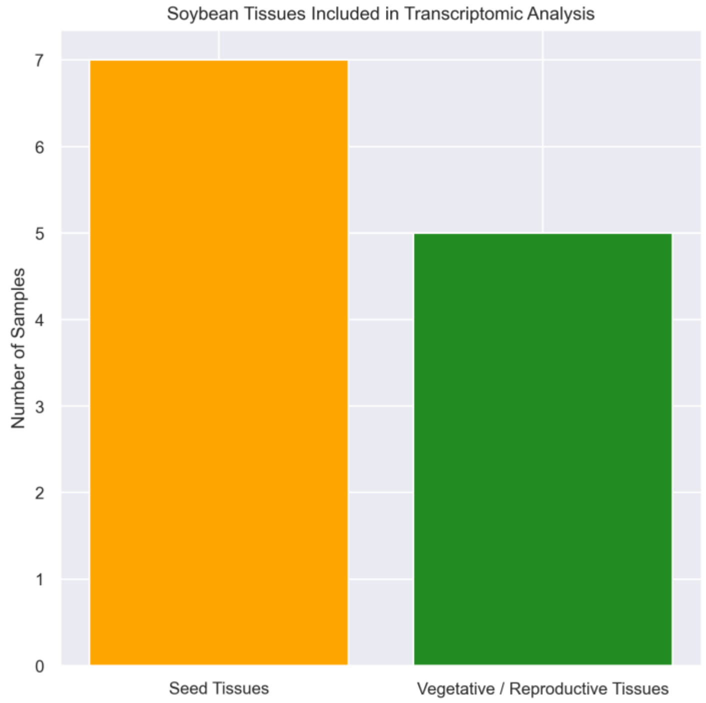

Supplementary Figure S1. Composition of soybean transcriptomic dataset. Bar plot showing the number of samples representing seed developmental stages and vegetative or reproductive tissues included in the analysis.

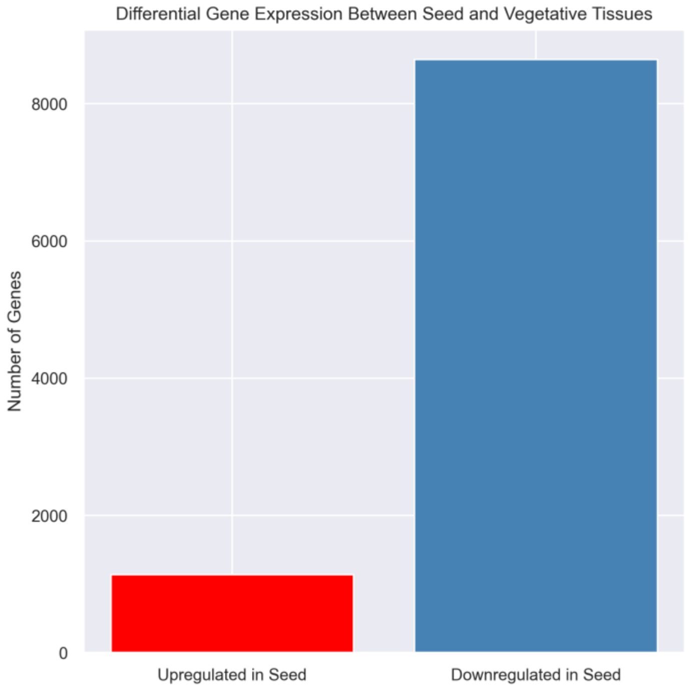

Supplementary Figure S2. Summary of differentially expressed genes. Bar plot showing the number of genes significantly upregulated and downregulated in seed tissues compared to non-seed tissues based on FDR and fold-change thresholds.
